# Supplementary material for: Silymarin Reduced Insulin Resistance in Non-Diabetic Women with Obesity
Source: Int J Mol Sci. 2024 Feb 8;25(4):2050. doi: 10.3390/ijms25042050 (PMC10888588; doi:10.3390/ijms25042050)
Supplement: Supplementary file 1 [file ijms-25-02050-s001.zip › ijms-2827530-supplementary.pdf]

International Journal of *Molecular Sciences*

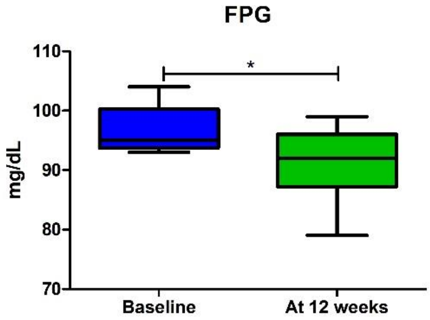

(**a**)

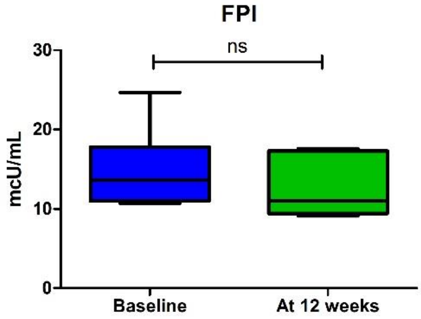

(**b**)

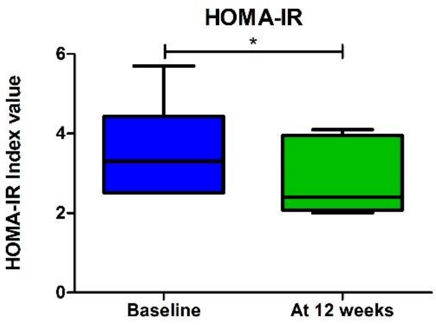

(**c**)

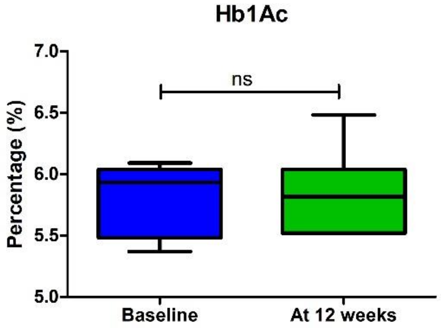

(**d**)

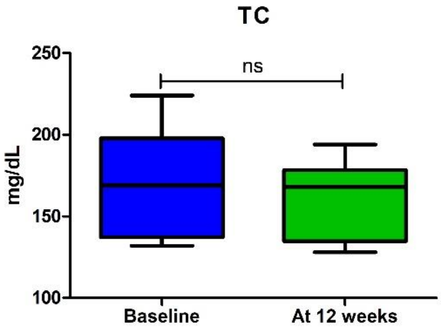

(**e**)

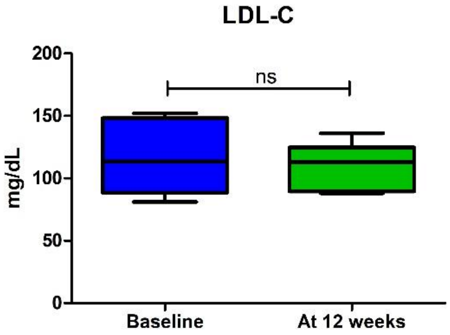

(**f**)

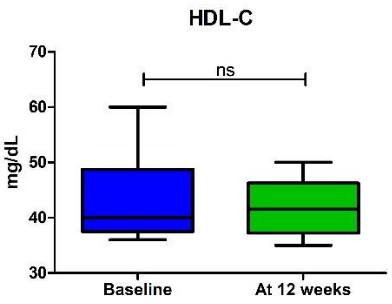
HDL-C
70
60
50
40
30
mg/dL
ns
Baseline
At 12 weeks

(**g**)

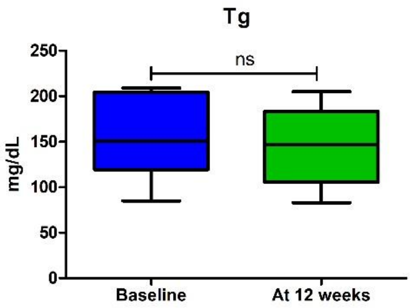
Tg
250
200
150
100
50
0
mg/dL
ns
Baseline
At 12 weeks

(**h**)

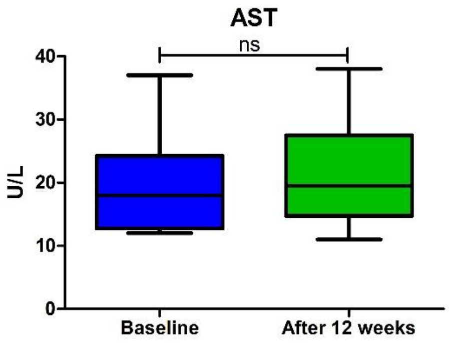
AST
40
30
20
10
0
U/L
ns
Baseline
After 12 weeks

(**i**)

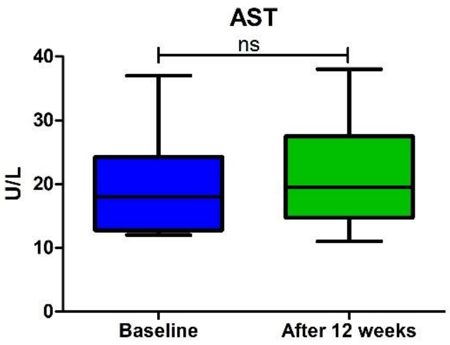
AST
40
30
20
10
0
U/L
ns
Baseline
After 12 weeks

(**j**)

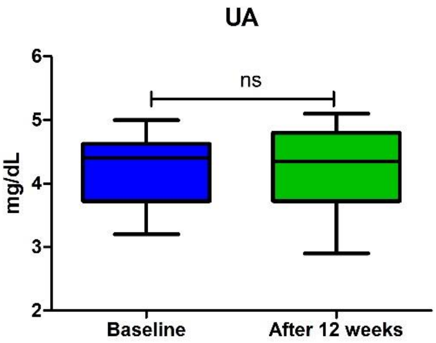
UA
6
5
4
3
2
mg/dL
ns
Baseline
After 12 weeks

(**k**)

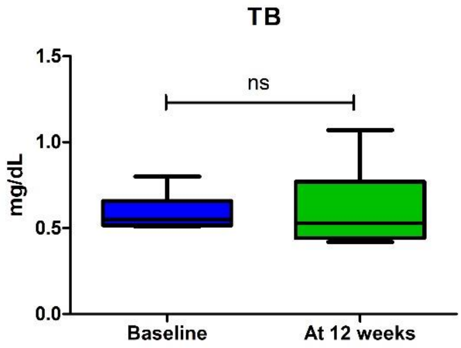
TB
1.5
1.0
0.5
0.0
mg/dL
ns
Baseline
At 12 weeks

(**l**)

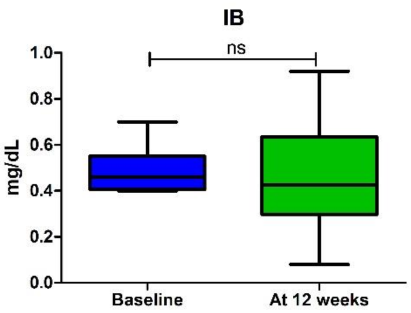

(**m**)

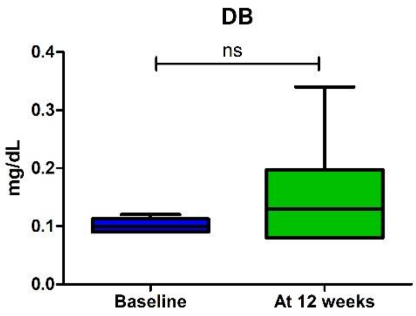

(**n**)

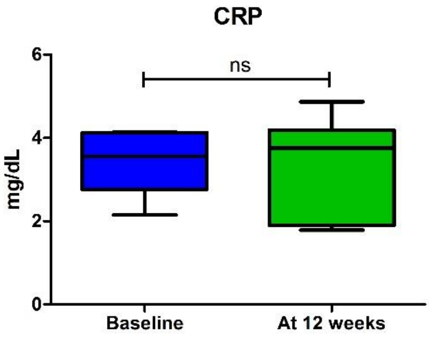

(**o**)

Figure S1. Biochemical parameters in patients at baseline (t = 0 weeks) and after silymarin treatment (t = 12 weeks) were calculated and compared using Student´s *t*-test ($n = 6$). The data are presented as the medians (interquartile ranges), * $p < 0.05$ was considered statistically significant. (**a**) fasting plasma glucose, (**b**) fasting plasma insulin, (**c**) HOMA-IR Index, (**d**) Hb1Ac, (**e**) total cholesterol, (**f**) low-density protein cholesterol, (**g**) high-density lipoprotein cholesterol, (**h**) triglycerides, (**i**) aspartate aminotransferase, (**j**) alanine aminotransferase, (**k**) uric acid, (**l**) total bilirubin, (**m**) indirect bilirubin, (**n**) direct bilirubin, and (**o**) C-reactive protein. Abbreviations: FPG, fasting plasma glucose; FPI, fasting plasma insulin; HOMA-IR, Homeostatic Model of the Insulin Resistance; Hb1Ac, glycated hemoglobin; TC, total cholesterol; LDL-C, low-density lipoprotein cholesterol; HDL-C, high-density lipoprotein cholesterol; TG, triglycerides; AST, aspartate aminotransferase; ALT, alanine aminotransferase; UA, uric acid; TB, total bilirubin; IB, indirect bilirubin; DB, direct bilirubin; CRP, C-reactive protein; ns, non-significant.

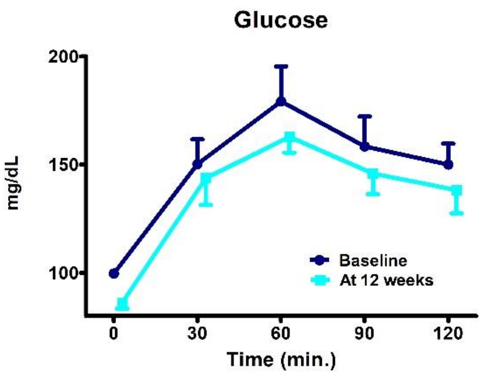

(**a**)

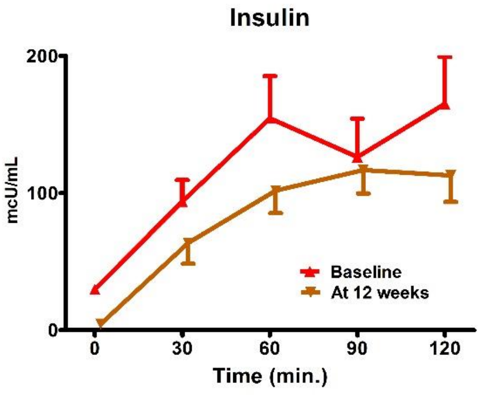

(**b**)

Figure S2. Glucose response (**a**), and insulin clearance (**b**) curves, expressed as area under the curve (AUC), during the oral glucose tolerance test (OGTT) at baseline (t = 0 weeks) and after silymarin treatment (t = 12 weeks). All data are expressed as the mean ± standard deviation (*n* = 6).
